# Novel urinary exosomal biomarkers of acute T cell-mediated rejection in kidney transplant recipients: A cross-sectional study

**DOI:** 10.1371/journal.pone.0204204

**Published:** 2018-09-18

**Authors:** Jeong-Hoon Lim, Chan-Hyeong Lee, Kyu Yeun Kim, Hee-Yeon Jung, Ji-Young Choi, Jang-Hee Cho, Sun-Hee Park, Yong-Lim Kim, Moon-Chang Baek, Jae Berm Park, Young-Hoon Kim, Byung Ha Chung, Sang-Ho Lee, Chan-Duck Kim

**Affiliations:** 1 Department of Internal Medicine, School of Medicine, Kyungpook National University, Kyungpook National University Hospital, Daegu, South Korea; 2 Department of Molecular Medicine, School of Medicine, Kyungpook National University, Daegu, South Korea; 3 Department of Surgery, Samsung Medical Center, Seoul, South Korea; 4 Division of Nephrology, Department of Internal Medicine, College of Medicine, Inje University, Pusan, South Korea; 5 Department of Internal Medicine, Seoul St. Mary’s Hospital, College of Medicine, The Catholic University of Korea, Seoul, South Korea; 6 Department of Internal Medicine, College of Medicine, Kyung Hee University, Seoul, South Korea; Universita degli Studi di Torino, ITALY

## Abstract

**Background:**

Acute rejection is hazardous to graft survival in kidney transplant recipients (KTRs). We aimed to identify novel biomarkers for early diagnosis of acute T cell-mediated rejection (TCMR) in urinary exosomes of KTRs.

**Methods:**

Among 458 graft biopsies enrolled in a cross-sectional multicenter study, 22 patients with stable graft function (STA) who had not shown pathologic abnormality and 25 patients who diagnosed biopsy-proven TCMR were analyzed. We performed proteomic analysis using nano-ultra performance liquid chromatography-tandem mass spectrometry (nano-UPLC-MS/MS) to identify candidate biomarkers for early TCMR diagnosis on urinary exosomes. We confirmed the protein levels of each candidate biomarker by western blot analysis.

**Results:**

A total of 169 urinary exosome proteins were identified by nano-UPLC-MS/MS. Forty-six proteins showed increased expression in STA patients, while 17 proteins were increased in TCMR patients. Among them, we selected five proteins as candidate biomarkers for early diagnosis of TCMR according to significance, degree of quantity variance, and information from the ExoCarta database. We confirmed the proteomic expression levels of five candidate biomarkers by western blot analysis in each patient. Of all candidate biomarkers, tetraspanin-1 and hemopexin were significantly higher in TCMR patients (STA:TCMR ratio = 1:1.8, *P* = 0.009, and 1:3.5, *P* = 0.046, respectively).

**Conclusions:**

Tetraspanin-1 and hemopexin were detected in KTR urine and could act as potential diagnostic proteins for TCMR.

## Introduction

Kidney transplantation (KT) is the treatment of choice for patients with end-stage renal disease [1]. Successful transplants improve quality of life and reduce the mortality risk, compared with patients undergoing chronic dialysis [2].

The development of more potent and effective immunosuppressive agents has decreased the acute rejection incidence in kidney transplant recipients (KTRs). However, there is no clear improvement in long-term graft survival [3, 4], and there exists no definitive diagnostic tool to assess this, apart from allograft biopsy [5].

Kidney allograft biopsy, coupled with histopathologic examination, is the gold standard for diagnosing acute rejection in KTRs; however, there exist several limitations. First, the procedure is invasive and can cause serious complications, such as bleeding and infection. Second, it is difficult to continuously monitor. Third, it is expensive. Last, histologic examination usually has excessive inter-observer disagreement on interpretation [6]. Early diagnosis of acute rejection is important for graft management and improving long-term graft survival; however, the aforementioned limitations influence the opportunity for early diagnosis.

Conventional indicators that reflect allograft function, such as serum creatinine, proteinuria, and color doppler sonography, are non-invasive, but are also non-specific for acute rejection diagnosis. Therefore, novel, non-invasive, sensitive, and specific diagnostic tools must be developed to help predict acute rejection.

Recent advances in omics technologies provide additional information about a disease and can help identify novel biomarkers. Based on these technologies, there are several studies that have attempted to identify diagnostic biomarkers for acute rejection in KTRs [7–10]. However, there has been no confirmed positive outcome as yet.

Therefore, this study aimed to employ a proteomics approach to identify novel biomarkers that could predict acute T cell-mediated rejection (TCMR) in a non-invasive manner.

## Materials and methods

### Study population and data collection

Four hundred fifty-eight graft biopsies, from 385 KTRs, were enrolled in the cross-sectional multicenter study ARTKT-1 (Assessment of immunologic Risk and Tolerance in Kidney Transplantation), which involved six university-based hospitals in Korea. Renal allograft biopsies were performed in patients undergoing acute clinical graft dysfunction or who were on protocol. The results of pathologic diagnosis were shown in S1 Table. In enrolled KTRs, 25 patients who diagnosed biopsy-proven acute TCMR and 22 patients with stable graft function (STA) who had not shown pathologic abnormality were selected. All blood and urine samples were collected at the time of biopsy, using an identical protocol, between August 2013 and July 2015. Estimated glomerular filtration rate (eGFR) was calculated using the Chronic Kidney Disease Epidemiology Collaboration (CKD-EPI) equation [11]. All patients provided written informed consent before inclusion. The study protocol was registered in the Clinical Research Information Service (CRIS Registration Number: KCT0001010), and was approved by the Institutional Review Board of each participating hospital. [Kyungpook National University Hospital (2013-10-010); Kyung Hee University Hospital at Gangdong; Kyung Hee University Hospital; Samsung Medical Center; St. Mary’s Hospital of the Catholic University of Korea; Inje University Busan Paik Hospital]. This study was conducted in accordance with the 2000 Declaration of Helsinki as well as the Declaration of Istanbul 2008.

### Definitions of STA and acute TCMR

STA was defined as patients with stable serum creatinine and absence of significant injury on graft biopsy [12]. Acute TCMR was diagnosed according to the Banff ‘07 classification [13]. The cases showed interstitial infiltration, tubulitis, and intimal arteritis.

### Urine exosome isolation in LC-MS analysis

Pooled urine from 22 STA and 25 TCMR patients was first centrifuged at 3,000 × *g* for 15 min at 4°C and then 17,000 × *g* for 15 min at 4°C to remove cells and cell debris. The resulting supernatant was subsequently centrifuged at 200,000 × *g* for 2 hours at 4°C. The pellet in each tube was resuspended in 50 μl isolation solution (250 mM sucrose/10 mM triethanolamine/0.5 mM PMSF/1 μM leupeptin), and then incubated with 60 mg/mL DTT for 10 min at 60°C to denature the zona pellucida domains in the Tamm-Horsfall protein (THP), thereby inhibiting aggregation and allowing THP to be removed from the supernatant. Crude exosomes were washed in PBS at 200,000 × *g* for 2 hours at 4°C and then resuspended in RIPA buffer [14].

### Urine exosome isolation for western blot analysis

Exosomes in 500 μl urine samples were concentrated using Amicon Ultra-15 (MWCO: 100 kDa, Millipore). The urine samples were centrifuged at 14,000 × *g* for 10 min at 4°C. To recover the concentrated urine, the filter device transferred and placed upside down in a clean centrifuge tube and centrifuged at 1,000 × *g* for 2 min at 4°C. Proteins were quantified using the Pierce BCA Protein Assay kit (Rockford, IL, USA).

### LC-MS sample preparation

Isolated urine exosomal proteins were resuspended in 100mM triethylammonium bicarbonate (TEABC; pH 8) containing 6 M Urea, 5 mM EDTA, and 2% SDS. The proteins were chemically denatured with 10 mM DTT for 20 min at 60°C, and alkylated with 50 mM iodoacetamide for 20 min at 25°C [15]. The denatured proteins were mixed with 30% acrylamide/bisacrylamide solution, 10% ammonium persulfate and tetramethylethylenediamine. The resulting gel was cut, then the gel was washed three times with 25 mM TEABC containing 50% acetonitrile (ACN). Trypsin digestion was performed in 25 mM TEABC overnight at 37°C. The peptides were extracted from the gel through exchange with two extraction buffers consisting of 0.1% formic acid (FA) in 25 mM TEABC or 0.1% FA in 50% ACN [16]. The buffer was dried in a SpeedVac and desalted by HLB cartridge (Waters, Milford, MA, USA).

### LC-MS analysis

LC-MS analysis was performed as described previously [15]. Resulting peptide was analyzed by nano-UPLC mass spectrometry using Q-Tof Premier (Waters, Manchester, UK). Peptides were injected into the trap column then resolved by nanoACQUITY C18 column (Waters). The peptides were resolved with a gradient of 3% to 45% CAN with 0.1% FA over 160 min at a 300 nL/min flow rate.

### Protein identification and quantification

The proteins were identified as described previously [15]. MS data were analyzed by MASCOT Distiller version 2.1 and MASCOT version 2.2.1 (Matrix Science, London, UK) using International Protein Index (IPI) HUMAN database version 3.78 (86,392 entries) [17]. The database was searched with a 0.5 Da fragment ion mass tolerance and a 0.2 Da parent ion tolerance. Two missed cleavages were allowed for trypsin digestion. Carbamidomethylation of cysteine and oxidation of methionine were considered variable modifications [18]. The false-discovery rate (FDR) was evaluated by repeated analysis using identical search parameters and validation criteria against a randomized decoy database created by MASCOT. The peptide were assigned if their ion scores were *P*<0.05. Proteins with more than two peptides were identified with confidence [15].

PEAKS 7 (Bioinformatics Solutions Inc., Waterloo, Canada) was used for Label-free protein quantification in triplicate. Total ion current (TIC) was used to convert all spectra to the same intensity range. Normalization with TIC encodes the average area under the peak. The quantification analysis was automatically normalized using TIC value provided by the PEAKS 7. Ion chromatography extraction was used to determine peptide abundance and the average abundance among the corresponding peptides was used to calculate the protein ratio. Protein ratios were regarded acceptable when the identified proteins contained one or more unique peptides [15].

### Candidate proteomic biomarker validation

Exosomal protein (20 μg) was separated by SDS-PAGE, transferred onto a nitrocellulose membrane, probed with each primary antibody, and incubated with horseradish peroxidase (HRP)-linked secondary antibody. Blots were visualized with enhanced chemiluminescence (ECL) detection reagents and quantified using ECL hyperfilm. Band volumes were measured by densitometry in at least three different experiments. Primary antibodies were used against the following proteins: tetraspanin-1 (TSPAN1) (H00010103, 1:500; Abnova), hemopexin (HPX) (ab124935, 1:500; Abcam), polymeric immunoglobulin receptor (PIGR) (ab91269, 1:500; Abcam), apolipoprotein A-I **(**APOA1) (ab52945, 1:500; Abcam), and lectin galactoside-binding soluble 3 binding protein (LGALS3BP) (ab123921, 1:500; Abcam).

### Functional protein association network analysis

For functional protein network analysis, we used Protein-protein interactions were predicted using the Search Tool for the Retrieval of Interacting Genes/Proteins (STRING) database v10.5 (http://www.string-db.org/) [19]. The minimum required interaction score was set to 0.4 (medium confidence) and the number of interacting proteins shown was set to a maximum of 20. The images of the networks were showed with confidence view settings in which line thickness indicates the strength of data support.

### Statistical analysis

The Kolmogorov-Smirnov tests were used to analyze distribution normality of measured variables. Data were summarized as mean value ± standard deviation, or median (interquartile range), or number and percentage (%) depending on the nature and distribution of the variables. Student's *t*-test or Mann-Whitney *U* tests were used to evaluate differences between the continuous variables. Pearson chi-square tests were applied to compare categorical variables. Receiver Operating Characteristic (ROC) analysis was performed to evaluate the potential of both TSPAN1 and HPX to discriminate between STA and acute TCMR. The performance was assessed by area under the curve (AUC). The statistical analysis was performed using SPSS 20.0 for Windows (SPSS Inc, Chicago, IL, USA). All *P* values were two-tailed, and *P*<0.05 was considered statistically significant.

## Results

### Patient characteristics

Table 1 describes the baseline demographic characteristics of the enrolled KTRs (22 with STA and 25 with acute TCMR). There were significant differences in the period since KT [STA vs. TCMR: 85.5 (16.0−175.0) days vs. 259.0 (84.0−696.0) days, *P* = 0.001], spot urine protein-to-creatinine ratio [0.05 (0.01−0.11) g/g vs. 0.21 (0.11−0.65) g/g, *P* = 0.001], and allograft function between STA and acute TCMR at the time of biopsy, including serum creatinine levels and eGFR (1.13 ± 0.35 mg/dL vs. 2.92 ± 1.90 mg/dL, *P*<0.001, and 74.9 ± 24.2 ml/min/1.73 m^2^ vs. 32.9 ± 18.9 ml/min/1.73 m^2^, *P*<0.001, respectively). However, no significant differences were observed in gender, age, and number of HLA mismatches between the two groups. Donor age was similar, and a living donor was more common in STA than in acute TCMR (77.3% vs. 44.0%, *P* = 0.02).

**Table 1:** Patient demographic and transplant characteristics.

Clinical characteristics	Stable graft function	Acute T cell-mediated rejection	*P* value
Number of patients	22	25	
Age (years)	44.2 ± 13.6	47.2 ± 11.8	0.177
Sex (% male)	12 (54.5)	16 (64.0)	0.510
Time since KT (days)	85.5 (16.0, 175.0)	259.0 (84.0, 696.0)	0.001
Serum creatinine (mg/dL)	1.13 ± 0.35	2.92 ± 1.90	<0.001
eGFR (ml/min/1.73 m^2^)	74.9 ± 24.2	32.9 ± 18.9	<0.001
Urine P/C ratio (g/g)	0.05 (0.01, 0.11)	0.21 (0.11, 0.65)	0.001
HLA mismatch	3.6 ± 1.7	3.5 ± 1.5	0.812
Donor age (years)	46.9 ± 11.3	44.8 ± 16.9	0.623
Donor gender, Male, n (%)	11 (50.0)	16 (66.7)	0.510
Donor source, Living, n (%)	17 (77.3)	11 (44.0)	0.020

Continuous variables are shown as mean ± standard deviation or median (interquartile range).

Abbreviations: KT, kidney transplantation; eGFR, estimated glomerular filtration rate; P/C, protein-to-creatinine.

### Identification and selection of STA and TCMR proteomic biomarkers

**Fig 1:**
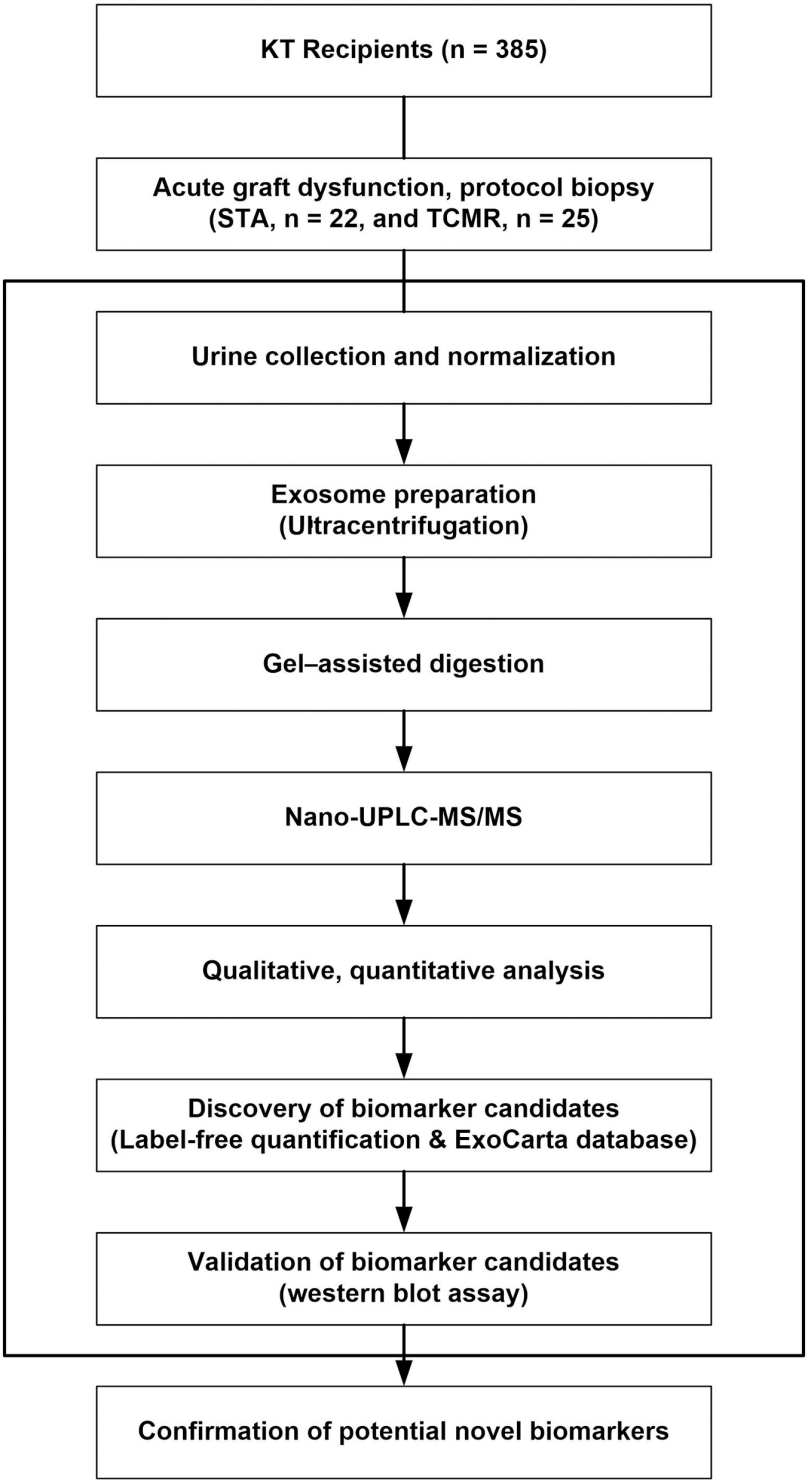
Flow diagram of the methods utilized for the analysis of urinary exosomes and discovery of candidate biomarkers. Abbreviations: STA, stable graft function; TCMR, T cell-mediated rejection; Nano-UPLC-MS/MS, nano-ultra performance liquid chromatography-tandem mass spectrometry.

**Fig 2:**
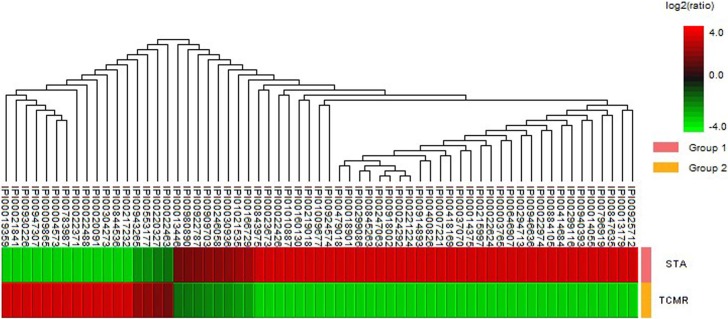
Heatmap demonstrating the level of common proteins in STA and acute TCMR. Abbreviations: STA, stable graft function; TCMR, T cell-mediated rejection.

The flow diagram for discovery and analysis of the urinary exosome biomarker is illustrated in Fig 1. To identify the exosomal proteins in pooled urine, we performed proteomic analysis by nano-UPLC-MS/MS. The total exosomal protein pool was evaluated using MASCOT in the IPI human sequence databases. Using high-confidence peptide sequences with an error rate less than 5%, we identified 138 and 100 proteins in the urine exosomes of STA and acute TCMR, respectively. Among them, 69 proteins were common to both STA and acute TCMR. Excluding the common proteins, 69 proteins were identified in STA only and 31 proteins in acute TCMR only. To verify the ratio of commonly identified proteins in each group, we performed protein quantification using the sum of normalized ion intensity expressed as relative values. Quantification analysis results are shown as a heatmap (Fig 2), which identifies 46 proteins upregulated in STA, and 17 proteins upregulated in acute TCMR. These proteins are listed along with a sample profile ratio between the two groups in Table 2.

**Table 2:** List of 63 upregulated proteins in STA or acute TCMR.

Accession	Significance (-10lg*P*)	STA Area	TCMR Area	Sample Profile (Ratio)	Description
**IPI00021841**	**11.12**	**0**	**6.28E+02**	**0:1.00**	**APOA1, Apolipoprotein A1**
IPI00783987	11.04	0	3.90E+02	0:1.00	C3, Complement C3 (Fragment)
IPI00247063	10.91	1.03E+03	0	1.00:0	NEP, Neprilysin
**IPI00022488**	**10.88**	**0**	**1.16E+03**	**0:1.00**	**HPX, Hemopexin**
**IPI00004573**	**10.88**	**0**	**1.29E+03**	**0:1.00**	**PIGR, Polymeric immunoglobulin receptor**
IPI00925712	10.8	8.36E+02	0	1.00:0	PROM1, Isoform 6 of Prominin-1
IPI00024292	10.68	9.91E+02	0	1.00:0	LRP2, Low-density lipoprotein receptor-related protein 2
IPI00845263	10.43	2.75E+02	0	1.00:0	FN1, fibronectin isoform 4 preproprotein
IPI00014055	10.28	1.65E+03	0	1.00:0	NAPSA, Napsin-A
**IPI00023673**	**10.26**	**3.66E+03**	**0**	**1.00:0**	**LGALS3BP, Lectin galactoside-binding soluble 3 binding protein**
IPI00221224	10.25	1.72E+03	0	1.00:0	ANPEP, Aminopeptidase N
IPI00304273	10.24	0	9.73E+01	0:1.00	APOA4, Apolipoprotein A-IV
IPI00215997	10.14	1.46E+03	0	1.00:0	CD9, CD9 antigen
IPI01009677	10.12	2.18E+02	0	1.00:0	ABCB1
IPI00009865	9.97	0	1.22E+03	0:1.00	KRT10, Keratin type I cytoskeletal 10
IPI00947307	9.97	0	3.15E+02	0:1.00	CP, cDNA FLJ58075 highly similar to Ceruloplasmin
IPI00847635	9.94	4.68E+02	0	1.00:0	SERPINA3, Isoform 1 of Alpha-1-antichymotrypsin
IPI01010887	9.79	7.45E+02	0	1.00:0	STOM, cDNA FLJ52062 highly similar to Erythrocyte band 7 integral membrane protein
IPI00014375	9.77	4.52E+02	0	1.00:0	ENPEP, Glutamyl aminopeptidase
IPI00844536	9.62	0	6.85E+02	0:1.00	RBP4
IPI00400826	9.61	1.42E+03	0	1.00:0	CLU, Isoform 2 of Clusterin
IPI00022426	9.36	1.32E+03	0	1.00:0	AMBP
IPI00914893	9.24	6.44E+02	0	1.00:0	SLC12A1, Isoform F of Solute carrier family 12 member 1
IPI00019359	8.86	0	6.13E+02	0:1.00	KRT9, Keratin type I cytoskeletal 9
IPI00924574	8.66	1.45E+03	0	1.00:0	APOD
IPI00299086	8.57	2.11E+03	0	1.00:0	SDCBP, Isoform 1 of Syntenin-1
IPI00843975	8.51	3.74E+02	0	1.00:0	EZR, Ezrin
IPI00007221	8.37	3.25E+02	0	1.00:0	SERPINA5, Plasma serine protease inhibitor
IPI00796919	8.36	2.84E+02	0	1.00:0	GLB1, Beta-galactosidase
IPI00022974	8.36	7.90E+02	0	1.00:0	PIP, Prolactin-inducible protein
IPI00479018	8.31	7.59E+02	0	1.00:0	SDCBP, Isoform 2 of Syntenin-1
IPI00918002	8.24	7.08E+02	0	1.00:0	MUC5B, Mucin 5AC oligomeric mucus/gel-forming
IPI00018901	8.24	5.37E+02	0	1.00:0	GGT1, Glutathione hydrolase 1 proenzyme
IPI00022624	8.19	1.07E+03	0	1.00:0	GPRC5A, Retinoic acid-induced protein 3
IPI00219018	8.06	4.39E+02	0	1.00:0	GAPDH, Glyceraldehyde-3-phosphate dehydrogenase
IPI00246058	7.94	3.40E+02	9.08E+01	1.00:0.27	PDCD6IP, Programmed cell death 6-interacting protein
IPI00909703	7.81	3.20E+02	8.58E+01	1.00:0.27	ANXA11
IPI00166729	7.71	6.57E+02	9.87E+01	1.00:0.15	AZGP1, Zinc-alpha-2-glycoprotein
IPI00922787	7.39	1.82E+02	6.18E+01	1.00:0.34	BROX, cDNA FLJ78829
IPI00980890	7.32	8.40E+02	3.32E+02	1.00:0.40	LAMP2, cDNA FLJ58780
IPI01021535	7.25	3.54E+02	5.75E+01	1.00:0.16	SLC44A4, Choline transporter-like protein 4 isoform 3
IPI00943265	7.1	6.73E+01	3.58E+02	1.00:5.32	IGKV4-1, Similar to Ig kappa chain V-IV region precursor
IPI00013446	6.35	8.78E+02	3.83E+02	1.00:0.44	PSCA, Prostate stem cell antigen
IPI00553177	6.29	1.80E+02	8.32E+02	1.00:4.63	SERPINA1, Alpha-1-antitrypsin
IPI00022463	6.26	7.85E+02	1.87E+03	1.00:2.38	TF, Serotransferrin
IPI00220327	6.26	4.04E+02	1.21E+03	1.00:3.01	KRT1, Keratin type II cytoskeletal 1
**IPI00030936**	**5.92**	**1.55E+03**	**3.68E+02**	**1.00:0.24**	**TSPAN1, Tetraspanin-1**
IPI00646907	5.72	1.54E+02	0	1.00:0	SLC12A3, Isoform 2 of Solute carrier family 12 member 3
IPI00418169	5.7	1.42E+02	0	1.00:0	ANXA2, Isoform 2 of Annexin A2
IPI00884105	5.61	2.63E+02	0	1.00:0	LAMP1, Lysosome-associated membrane glycoprotein 1
IPI00003765	5.58	1.11E+02	0	1.00:0	CAPN7, Calpain-7
IPI00946636	5.54	1.40E+02	0	1.00:0	ATP6V1A, cDNA FLJ51804
IPI00020091	5.52	0	1.12E+02	0:1.00	ORM2, Alpha-1-acid glycoprotein 2
IPI00940393	5.51	3.85E+02	0	1.00:0	EEF1A1
IPI00013179	5.49	7.22E+02	0	1.00:0	PTGDS, Prostaglandin-H2 D-isomerase
IPI00160130	5.43	7.75E+00	0	1.00:0	CUBN, Cubilin
IPI00217232	5.42	0	3.40E+02	0:1.00	SUCLA2, Isoform 2 of Succinyl-CoA ligase [ADP-forming] subunit beta mitochondrial
IPI00294713	5.4	8.29E+02	0	1.00:0	MASP2, Isoform 1 of Mannan-binding lectin serine protease 2
IPI00930226	5.39	0	2.86E+02	0:1.00	ACTG1, cDNA FLJ57283
IPI00414684	5.38	2.71E+02	0	1.00:0	SEMG1, Isoform 2 of Semenogelin-1
IPI00037070	5.37	2.88E+02	0	1.00:0	HSPA8
IPI00022371	5.31	0	5.59E+02	0:1.00	HRG, Histidine-rich glycoprotein
IPI00299116	5.29	1.75E+03	0	1.00:0	PODXL, Podocalyxin-like isoform 2 precursor

Proteins highlighted in bold were selected as biomarker candidates.

Abbreviations: STA, stable graft function; TCMR, T cell-mediated rejection.

### Candidate STA and TCMR proteomic biomarker validation

To discover specific exosomal proteins in each group, we chose candidate proteins based on the proteomic analysis. A total of 63 proteins were identified through protein quantification using label-free LC-MS/MS of urinary exosomes. Candidate biomarkers were selected from amongst these proteins according to several filtration conditions, as follows: (1) proteins had high significance; (2) proteins were known to be present in extracellular vesicles using the Exocarta database; (3) proteins were mentioned in references related to acute graft rejection. Finally, we selected five candidate proteins that were predicted to differ between STA and acute TCMR. The identified proteins were: TSPAN1, HPX, PIGR, APOA1, and LGALS3BP.

**Fig 3:**
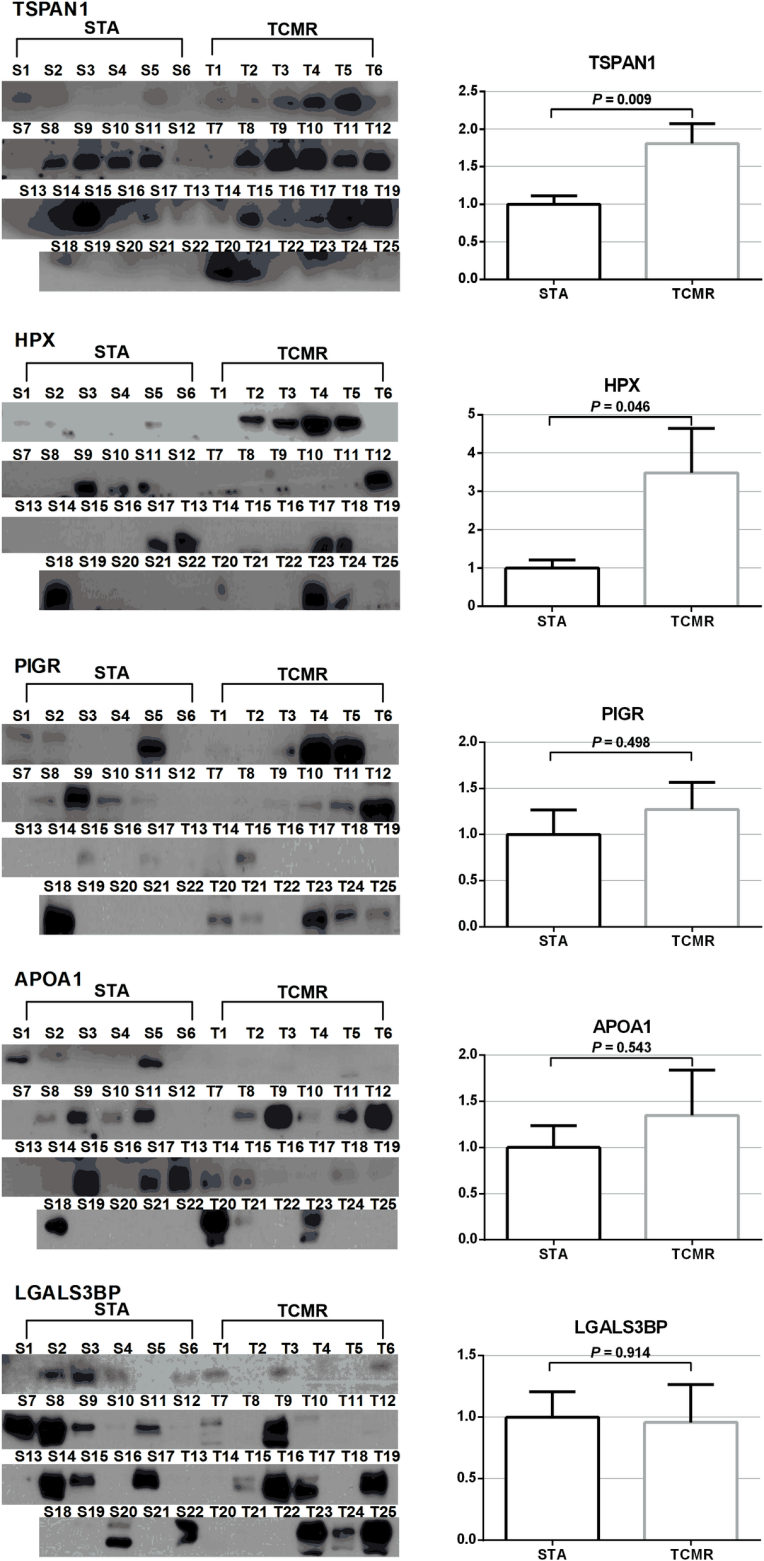
Validation of identified biomarker candidates. Left column displays the western blot assay results. Right column indicates the comparison of intensities from the western blot assay. Abbreviations: STA, stable graft function; TCMR, T cell-mediated rejection; TSAN1, tetraspanin-1; HPX, hemopexin; PIGR, polymeric immunoglobulin receptor; APOA1, apolipoprotein A-I; LGALS3BP, lectin galactoside-binding soluble 3 binding protein.

In order to validate the selected candidate biomarker expression, we performed western blot analysis of individually pooled urine. TSPAN1 and HPX expression levels were significantly higher in acute TCMR patients compared to STA patients [STA:acute TCMR ratio (Ratio) = 1:1.81, *P* = 0.009, and 1:3.48, *P* = 0.046, respectively) (Fig 3). However, the other candidate biomarkers, PIGR (Ratio = 1:1.27, *P* = 0.50), APOA1 (Ratio = 1:1.35, *P* = 0.54), and LGALS3BP (Ratio = 1:0.96, *P* = 0.91) showed no significant difference.

**Fig 4:**
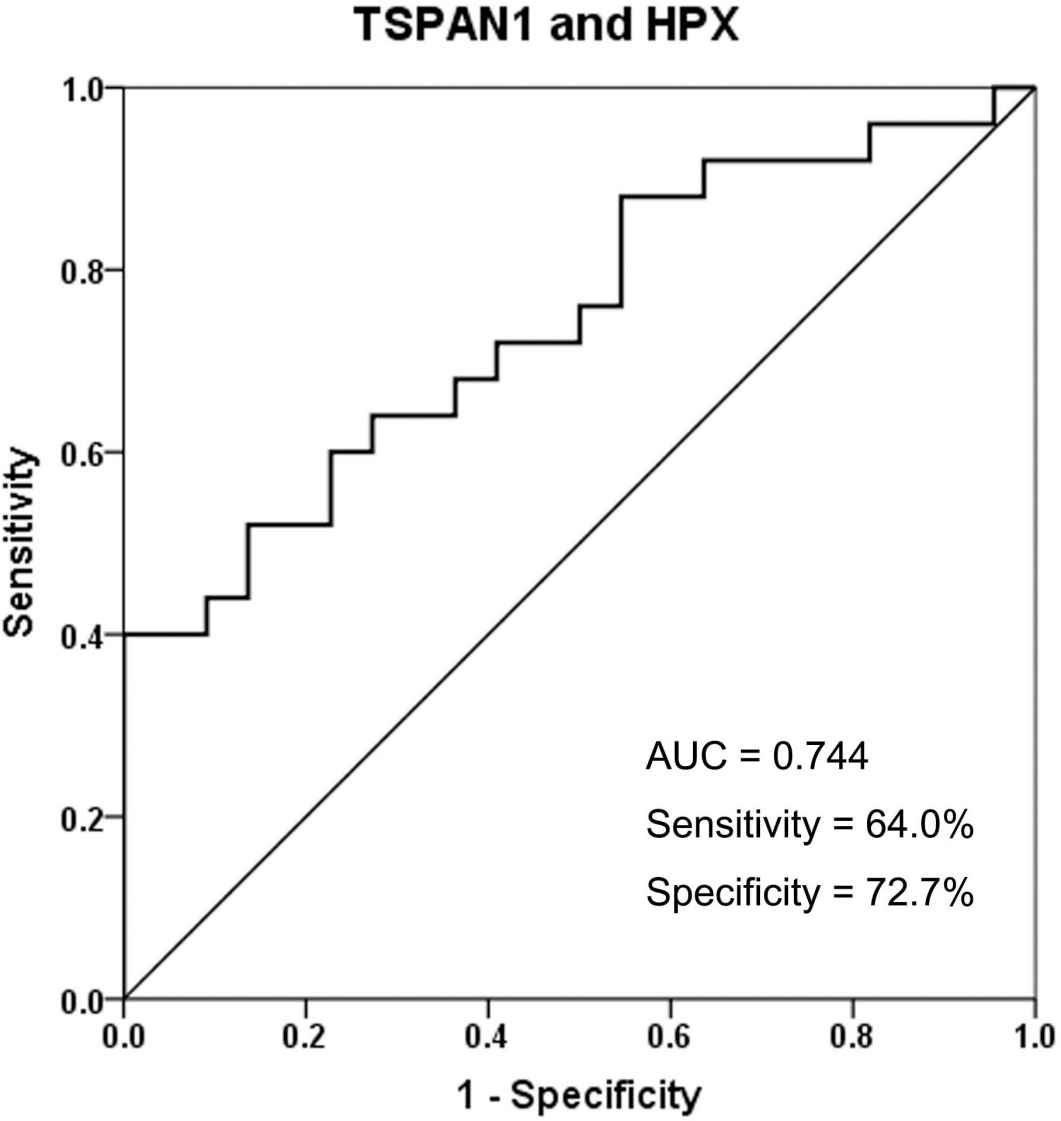
ROC curve for the differentiation between STA and acute TCMR. Abbreviations: STA, stable graft function; TCMR, T cell-mediated rejection; TSPAN1, tetraspanin-1; HPX, hemopexin.

ROC analysis was subsequently used to evaluate the potential of both TSPAN1 and HPX to discriminate between STA and acute TCMR (Fig 4). The resulting AUC was 0.744, while sensitivity and specificity were 64.0% and 72.7%, respectively.

### Analysis of functional interaction networks for the validated proteomic biomarkers

**Fig 5:**
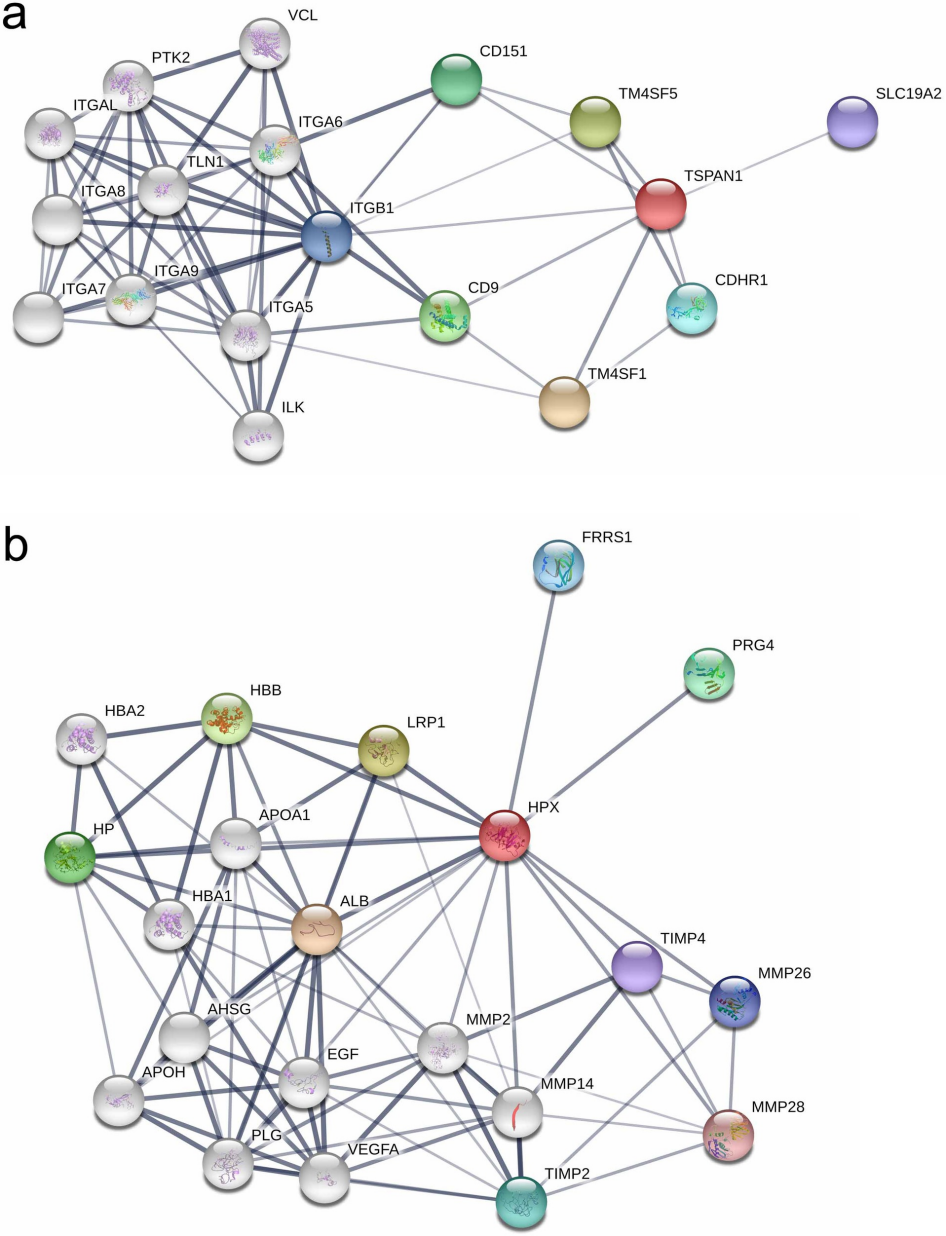
**Protein-protein interaction networks of tetraspanin-1 (a) and hemopexin (b).** Functional protein-protein interactions were constructed using the Search Tool for the Retrieval of Interacting Genes/Proteins (STRING) database. Line thickness indicates the strength of data support for protein-protein interaction. Colored nodes indicate the query proteins and first shell of interactors; White nodes indicate the second shell of interactors.

After validation, we used the STRING database to predict functional protein–protein interactions. The two validated proteins, TSPAN1 and HPX, were used for interaction analysis and experimentally derived interactions were utilized to depict the respective interactomes (Fig 5). In the TSPAN1 network map, integrin alpha, beta subunits, and other transmembrane 4 superfamily proteins were suggested to interact with TSPAN1. In the HPX network map, albumin, growth factors, matrix metalloproteinases (MMPs), and tissue inhibitor of metalloproteinases (TIMPs) were suggested to interact with HPX.

## Discussion

Development of a non-invasive monitoring method that can predict acute rejection in advance and distinguish acute rejection from other causes of allograft injury, such as BK virus nephropathy and calcineurin inhibitor toxicity, has shown increased urgency following the numerical increment of KT and prolonged life expectancy of KTRs. Acute rejection plays a critical role on graft survival [20], and occurs at an approximately 10% rate at most transplant centers, in spite of improved immunosuppressants [21, 22]. Regular serum creatinine and proteinuria level monitoring has been commonly used to predict acute rejection, but these are non-specific biomarkers and detectable differences generally indicate an allograft has already established irreversible injury. Allograft biopsy is an alternate diagnostic tool, but is invasive and cannot be used as a serial monitoring method, although it is considered the gold standard for transplant injury detection [12]. For these reasons, we aimed to identify novel biomarkers that could specifically diagnose acute TCMR in KTRs in a non-invasive manner.

Urine has been known as a valuable source of molecules capable of acting as diagnostic markers for renal disease. In particular urinary exosomes, which express 1,132 proteins, including several disease-related proteins, have been reported as appropriate source material for discovering de novo candidate biomarkers [23–25]. To identify potential biomarkers, we utilized nano-UPLC-MS/MS, which has several advantages over conventional LC-MS/MS, such as: (1) it allows peptide mixture analysis in sample-limited situations (e.g. proteolytically digested proteins isolated by two-dimensional gel electrophoresis); (2) there is a large decrease in mobile and stationary phase consumption, including toxic reagents; and (3) it couples easily to mass spectrometry [26, 27]. Through nano-UPLC-MS/MS, we initially identified five candidate biomarkers, after which we validated these in individual urinary exosomes using the western blot assay. In general, the strength of mass spectrometry is in protein identification, not quantification [25]. Therefore, it is likely that biomarker validation through protein abundance is more efficient when using an antibody-based assay, rather than by quantitative mass spectrometry [25]. Using this approach, we demonstrated TSPAN1 and HPX could act as potential biomarkers for acute TCMR.

The tetraspanin family comprises cell surface glycoproteins containing four transmembrane domains and two conserved extracellular loops [28]. Tetraspanins are implicated in various fundamental cellular processes including cell adhesion and migration, as well as intracellular signaling and trafficking [29]. They also influence and control diverse roles in immunity [29]. As shown in Fig 4, TSPAN1 is closely associated with various integrins. Integrins are known to play important roles in T cell-mediated immune responses by regulating the T cell and antigen presenting cell circulatory behavior [30, 31]. Furthermore, they promote of T cell rolling and adhesion, and direct cell trafficking and retention within peripheral tissue [31]. We speculate that through these interactions with integrins, increased TSPAN1 expression could be linked with acute TCMR.

HPX, also known as Beta-1B-glycoprotein, is a positive acute phase reactant with an anti-inflammatory action [32]. HPX is present in mammalian and human circulation, and is predominantly synthesized in the liver [33]. In addition, HPX is produced by tumor necrosis factor alpha-stimulated human mesangial cell and acts as a potential proteinuria-promoting factor associated with the corticosteroid-responsive nephrotic syndrome [34]. HPX interactions with other proteins are largely divided into two groups. One group is related to inflammation-related proteins, such as albumin, alpha-2-HS-glycoprotein (AHSG), and cellular growth factors. The other group is related to MMPs and TIMPs. Increased HPX might also be associated with decreased levels of negative acute phase reactants, such as albumin and AHSG, and increased levels of growth factors, such as epidermal growth factor and vascular endothelial growth factor A, which are indicative of up-regulated inflammation [35, 36]. MMPs influence the progression of inflammation via leukocyte recruitment, processing chemokines and cytokines, and pathogen clearance [37–40]; TIMPs are their natural inhibitors [37]. The relationships with these proteins would have resulted in higher HPX expression in acute TCMR patients.

Several studies have identified multiple protein biomarkers that are abundant in KTRs with acute rejection [41, 42]. However, to the best of our knowledge, this is the first study which identified novel potential proteomic biomarkers associated with acute rejection, especially in Asian KTRs. The incidence of acute rejection, immunosuppressive concentration, and graft survival are notably different according to racial differences [43–45]. Thus, developing an ethnologically customized diagnostic tool is important for KTRs.

Although our study sample size was small, there is certainly no simple rule of thumb to determine the necessary sample size for the omics study to find novel biomarkers. However, rejection is a heterogeneous process. Although we applied stringent histopathologic criteria to define acute TCMR, a larger sample size might be necessary to cover the broad spectrum of TCMR. In addition, the TSPAN1 quantification results from label-free quantification and western blot assay were opposite. It would be related to differences in the way samples were used and in analysis methods. We used pooled urine samples in LC-MS/MS analysis and individual urine samples in western blot assay.

In conclusion, we identified several biomarker candidates for acute TCMR from clinical urinary exosomes using LC-MS/MS. Subsequent validation of the proteomic discoveries by western blot assay confirmed that TSPAN1 and HPX could act as potential diagnostic proteins for acute TCMR. To demonstrate the clinical effectiveness of TSPAN1 and HPX, appropriately powered clinical trials with a sufficient number of TCMR and control patients, as well as a sufficient study period are deemed necessary in the near future.

## Supporting information

S1 Table Pathologic diagnosis for enrolled patients. (DOCX)

S2 Table Western blot results of biomarkers. (DOCX)
