# Supplementary material for: Novel urinary exosomal biomarkers of acute T cell-mediated rejection in kidney transplant recipients: A cross-sectional study
Source: PLoS One. 2018 Sep 18;13(9):e0204204. doi: 10.1371/journal.pone.0204204 (PMC6143249; doi:10.1371/journal.pone.0204204)
Supplement: S1 Table — (DOCX) [file pone.0204204.s001.docx]

**Supplemental Table 1. Pathologic diagnosis for enrolled patients**

| Diagnosis | n (%) |
| --- | --- |
| Normal | 142 (31.0) |
| TCMR | 76 (16.6) |
| Suspicious for TCMR | 55 (12.0) |
| ABMR | 64 (14.0) |
| Acute tubular necrosis | 31 (6.8) |
| Glomerulonephritis | 29 (6.3) |
| CNI toxicity | 26 (5.7) |
| BK virus nephropathy | 13 (2.8) |
| Others^*^ | 22 (4.8) |

^*^Others included diabetic nephropathy, acute interstitial nephritis, hypertensive nephrosclerosis, and nonspecific interstitial fibrosis and tubular atrophy.

Abbreviations: TCMR, T cell-mediated rejection; ABMR, antibody-mediated rejection; CNI, calcineurin inhibitor.
