# Supplementary material for: Novel urinary exosomal biomarkers of acute T cell-mediated rejection in kidney transplant recipients: A cross-sectional study
Source: PLoS One. 2018 Sep 18;13(9):e0204204. doi: 10.1371/journal.pone.0204204 (PMC6143249; doi:10.1371/journal.pone.0204204)
Supplement: S2 Table — (DOCX) [file pone.0204204.s002.docx]

**Supplemental Table 2. Western blot results of biomarkers**

| **Sample** | **Tetraspanin-1** | **Hemopexin** |
| --- | --- | --- |
| STA-1 | 2.013 | 1.1083 |
| STA-2 | 1.4679 | 2.0842 |
| STA-3 | 0.3563 | 0.5589 |
| STA-4 | 0.3823 | 0.4202 |
| STA-5 | 1.1431 | 1.1626 |
| STA-6 | 0.6378 | 0.6657 |
| STA-7 | 0.4207 | 0.4520 |
| STA-8 | 1.1270 | 0.7272 |
| STA-9 | 1.7029 | 2.2544 |
| STA-10 | 1.3001 | 1.2039 |
| STA-11 | 1.2006 | 1.1025 |
| STA-12 | 0.2487 | 0.2600 |
| STA-13 | 0.5332 | 0.6152 |
| STA-14 | 1.0404 | 0.4878 |
| STA-15 | 2.1783 | 0.2264 |
| STA-16 | 0.7194 | 0.1342 |
| STA-17 | 0.5287 | 3.5363 |
| STA-18 | 1.1449 | 3.3247 |
| STA-19 | 0.6850 | 0.5931 |
| STA-20 | 0.9487 | 0.4568 |
| STA-21 | 0.9410 | 0.3452 |
| STA-22 | 1.2804 | 0.2803 |
| TCMR-1 | 1.3397 | 0.6311 |
| TCMR-2 | 1.4428 | 8.2783 |
| TCMR-3 | 2.0058 | 11.9226 |
| TCMR-4 | 2.6488 | 23.8704 |
| TCMR-5 | 4.3519 | 15.1779 |
| TCMR-6 | 0.9961 | 0.4902 |
| TCMR-7 | 0.4765 | 0.2568 |
| TCMR-8 | 1.3721 | 0.3963 |
| TCMR-9 | 2.1967 | 0.5404 |
| TCMR-10 | 2.0954 | 0.0358 |
| TCMR-11 | 1.1852 | 0.2627 |
| TCMR-12 | 1.6322 | 3.0673 |
| TCMR-13 | 0.2306 | 6.1623 |
| TCMR-14 | 0.2930 | 0.2080 |
| TCMR-15 | 0.8181 | 1.0163 |
| TCMR-16 | 0.7322 | 0.9582 |
| TCMR-17 | 0.8103 | 5.3506 |
| TCMR-18 | 1.4542 | 3.851742 |
| TCMR-19 | 1.3236 | 0.1809 |
| TCMR-20 | 5.9950 | 0.4298 |
| TCMR-21 | 3.7128 | 0.0484 |
| TCMR-22 | 2.1327 | 0.1038 |
| TCMR-23 | 3.0069 | 2.9148 |
| TCMR-24 | 1.8035 | 0.6963 |
| TCMR-25 | 1.1144 | 0.1623 |

Abbreviations: STA, stable graft function; TCMR, T cell-mediated rejection.
